# Respiratory Cancer and Non-Malignant Respiratory Disease-Related Mortality among Older Construction Workers-Findings from the Health and Retirement Study

**DOI:** 10.4172/2329-6879.1000235

**Published:** 2016-05-30

**Authors:** Xuanwen Wang, Xiuwen Sue Dong, Laura Welch, Julie Largay

**Affiliations:** CPWR-The Center for Construction Research and Training, United States

**Keywords:** Respiratory cancer, Non-malignant respiratory disease, Mortality, Construction workers, Smoking

## Abstract

**Objective:**

This study explored the risk of respiratory cancer and non-malignant respiratory disease (NMRD)-related mortality among older construction workers.

**Methods:**

Analyzed data from the 1992–2010 RAND Health and Retirement Study (HRS) and the HRS National Death Index – Cause of Death file. About 25,183 workers aged 50 years and older were examined, including 5,447 decedents and 19,736 survivors, of which 1,460 reported their longest job was in construction. Multinomial logistic regression assessed the differences in mortality between workers’ longest occupations, controlling for confounders.

**Results:**

After adjusting for smoking and demographics, construction workers were almost twice as likely to die from respiratory cancer (OR = 1.65; CI: 1.10–2.47) or NMRD (OR = 1.73; CI: 1.16–2.58) compared to white-collar workers.

**Conclusions:**

This study adds to the growing evidence that respiratory cancer and NMRD are frequently associated with construction exposure.

## Introduction

Construction workers are exposed to a range of occupational exposures, including a number of known respiratory hazards such as asbestos [1–3], silica [4,5], and welding fumes [6–8]. Exposure to asbestos, silica, and the mixture of dust, fumes, gases, and vapors as well as smoking, can lead to chronic obstructive pulmonary disease (COPD) [9,10]. Toren et al. [11] followed a large cohort of Swedish construction workers from 1971 to 2011 and found that workers with any occupational exposure to dust, fumes, gases, and vapors showed an increased mortality due to COPD. Some of the studies among construction workers have found a significant excess of pneumoconioses specifically, or non-malignant respiratory diseases (NMRD) overall [12–15]. Hutchings [16] and Rushton [17] assessed the burden of occupational cancer in Great Britain, and estimated that 16.5% of lung cancers were attributable to occupational exposure to known human carcinogens as defined by the International Agency for Research on Cancer (IARC); this increased to 21.6% if exposure to suspected human carcinogens was also included. In addition, it was found that half of all lung cancers among construction workers were attributable to occupational exposures in which 50% were caused by asbestos, and another 25% were attributed to silica exposure.

Although asbestos use differed between Great Britain and the U.S. in terms of peak years when used and industrial applications, similar results were found among U.S. construction workers. Data from the Building Trades National Medical Screening Program (BTMed) showed that U.S. construction workers who worked in the Department of Energy sites had a noticeably higher prevalence of abnormal chest x-rays and pulmonary function tests than those employed in administrative or support positions, which was consistent with their exposure levels to workplace hazards [18]. A number of previous cohort studies reported either an elevated proportional mortality ratio or an elevated standard mortality ratio for lung cancer among all construction workers or in a specific construction trade [12–15,19–27]. Excess risk of lung cancer has been reported for cement masons, roofers, operating engineers, laborers, electricians, ironworkers, carpenters, plumbers, brick masons, and sheet metal workers. However, only a few of these studies have been able to adjust for smoking; those that could adjust for smoking have the potential for participation bias with the cohort recruited into a medical surveillance program [24,25,27]. Prior studies have also reported an excess of NMRD among construction workers. Asbestosis and silicosis were well-recognized hazards for a number of the construction trades [18].

Despite a great number of studies, most of them were based on one occupation [28,29] or a particular worksite [11,18,25]. As a result, questions remain about the magnitude of the risk for both malignant and non-malignant respiratory disease in the U.S. construction industry. Moreover, respiratory diseases, including lung cancer, are known to have many causes and long latency periods. These characteristics pose serious challenges for identifying occupationally induced respiratory diseases, in particular for mobile construction workers with a high prevalence of current and former smokers [18]. Using longitudinal survey data from the Health and Retirement Study on the U.S. population aged 50 years and older, a recent study found that the prevalence of lung disease more than doubled among the older construction worker cohort in a 10-year follow-up period [30]. The study indicated that the prevalence of lung diseases among workers in construction trades in the follow-up period was significantly higher than those in white-collar occupations, suggesting that lung diseases due to construction exposures may not emerge until later in life. However, the study only examined chronic diseases and did not cover mortality. To further understand the risk of both malignant and non-malignant respiratory diseases, this study examined respiratory cancer- and NMRD-related mortality among older construction workers.

## Methods

### Data sources

This study analyzed two datasets: the RAND Health and Retirement Study (HRS) dataset [31,32] and the HRS cross-year National Death Index (NDI) - Cause of Death file. The HRS, a nationally representative longitudinal survey of U.S. residents aged 50 years and older, is sponsored by the National Institute on Aging (grant number NIA U01AG009740) and is conducted by the University of Michigan. The initial sample of HRS in 1992 contained 12,652 people who were born between 1931 and 1941. Several age cohorts have since been added to the HRS in the waves following 1992. The survey is conducted every two years and contains detailed information on demographics, employment history, health conditions, and health behaviors. The RAND HRS data files were produced by the RAND Center for the Study of Aging, using data cleaning and processing for maximum comparability across waves. The HRS cross-year NDI – Cause of Death file was obtained through a data use agreement with the University of Michigan. Ten waves of the RAND HRS data for all age cohorts between 1992 and 2010 were linked to the 1992–2011 NDI - Cause of Death file to identify the year and cause of death of older workers. To modulate possible healthy worker effects, respondents who did not report their longest occupation were excluded. The total sample size used in this study was 25,183, including 5,447 decedents and 19,736 survivors. Detailed descriptions of the HRS data, such as sampling, weights, and definitions of variables in each wave, are publicly accessible and can be found on the HRS website, http://hrsonline.isr.umich.edu/.

### Terms and definitions

#### Outcome Measure

*Cause of mortality* was based on the International Classification of Diseases, Ninth Revision (ICD-9) and Tenth Revision (ICD-10). Deaths recorded in the NDI file prior to 1999 were coded using the ICD-9, while deaths occurring in 1999 and thereafter were coded using the ICD-10. Respiratory disease-related mortality includes Respiratory cancer (ICD-9 codes: 160–165; ICD-10 codes: C30–C39) and NMRD (ICD-9 codes: 460–519; ICD-10 codes: J00–J99). Other disease-related mortality includes any diseases other than respiratory cancer and NMRD.

#### Occupational Exposure

*Construction workers* include respondents who reported that their longest job was in construction or extraction trades as well as those whose longest job was in other blue-collar occupations (e.g., mechanics or repair personnel, operators) and whose longest industry (Although Construction and Mining were included in the same group in HRS masked industry for years 1992–2004, Mining only accounted for less than 10% of the group. Therefore, Mining was not separated from Construction in this study) was in construction. *Non-construction workers* include respondents whose longest job was in other blue-collar occupations and their longest industry was not in construction. Managerial and professional occupations, clerical, and administrative support were combined as *white-collar workers. Longest-job tenure* is the respondent’s years of tenure on the longest-held job reported. The longest job may be the current job, a past job held since the first HRS interview, or a job reported in the job history data.

#### Health Conditions and Health Behaviors

*Perceived physical health* was regrouped into three categories (i.e., excellent, very good/good, and fair/poor) from the five original categories in the dataset. *Mental health* was measured by the Center for Epidemiologic Studies Depression (CES-D) score, ranging from 0 to 8; the lower the CES-D score, the better the mental health. The CES-D score was the sum of six “negative” indicators and two “positive” indicators. The six negative indicators measured whether the respondent experienced the following sentiments all or most of the time (1 = Yes; 0 = No): depression, everything was an effort, sleep was restless, felt alone, felt sad, and could not get going. The two positive indicators measured whether the respondent felt happy and enjoyed life, all or most of the time (0 = Yes; 1 = No). The number of *diagnosed health conditions* was the summation of positive responses to eight questions in the form, “Has a doctor ever told you that you have…?” Respondents were asked about high blood pressure, diabetes, cancer, lung disease, heart disease, stroke, psychiatric problems, and arthritis.

Respondents’ *body mass index* (BMI) was categorized as underweight (BMI < 18.5), normal weight (BMI = 18.5–24.9), overweight (BMI = 25.0–29.9), or obese (BMI ≥ 30). *Smoking status* includes “never smoker” (never smoked cigarettes in their lifetime); “former smoker” (smoked cigarettes in their lifetime, but were not currently smoking); and “current smoker.” *Alcohol consumption* was measured by asking respondents to estimate the number of drinks they had on days they consumed alcohol in the last three months, ranging from non-drinker to five or more drinks per day.

*Physical activity* was measured by asking respondents about the frequency of vigorous physical activity, including sports, heavy housework, or physical labor on the job. *Physical inactivity* was defined as participating in vigorous activity less than three times per week. For the decedents, all of the measures except for age were obtained one to two years prior to the mortality outcome assessment; age for decedents was determined at the time of death. For the survivors, all the measures were from 2010.

### Data analysis

The unadjusted odds ratios of respiratory cancer and NMRD-related mortality were calculated by workers’ longest occupation, demographics, health conditions, and health behaviors using univariate logistic regressions. A multinomial logistic regression model [33,34] was applied to assess the differences in mortality between workers’ longest occupation, adjusted for the effects of age, race/ethnicity, marital status, number of diagnosed health conditions, BMI, and smoking. SAS (version 9.4) survey procedures, which account for the complex, multi-stage sampling design of the HRS, were used to conduct the data analyses. The sample weights, primary sampling unit markers, and strata markers of the HRS, were applied in all data analyses according to the survey design. Therefore, all results are based on weighted numbers.

## Results

Table 1 summarizes overall mortalities in this study sample by decedents’ longest occupation and cause of mortality. Between 1992 and 2011, 383 (weighted number 1,118, 156) older construction workers died from any cause. The proportion of respiratory cancer-related deaths (respiratory and intra-thoracic organs) for construction workers was nearly twice that for white-collar workers in this sample (14.6% versus 8.3%). Construction workers also had a higher percentage of deaths from diseases of the respiratory system (NMRD) than their white-collar counterparts (13.4% versus 8.9%).

Table 2 provides sample characteristics and unadjusted odds ratios of mortality from respiratory cancer and NMRD among workers by their longest occupation and major demographic characteristics. The average age of the sample, including both decedents and survivors, was 66.5 years. About 6% of the sample reported their longest job was in construction trades or blue-collar occupations in the construction industry.

The unadjusted odds ratios for respiratory cancer and NMRD mortalities among construction workers were 2.35 and 2.04, respectively, more than twice the odds of white-collar workers. Older, male, less educated, and separated/divorced/widowed workers had a higher odds of dying from respiratory diseases than their corresponding counterparts. No significant associations were found between the respiratory mortality and longest job tenure and geographic regions.

The unadjusted odds ratios of respiratory mortality among workers by health conditions and health behaviors are presented in Table 3. Workers who died of respiratory cancer or NMRD were more likely to have poor physical or mental health before death. The odds of dying from respiratory cancer among workers who reported fair or poor physical health one to two years prior to death, were six times higher than those who had excellent or very good health. The unadjusted odds ratio for respiratory cancer among workers who had worse mental health (CES-D score = 3–8) was 3.35, compared to those who reported the best mental health (CES-D score = 0). On average, workers reported two doctor-diagnosed health conditions. Older workers who had one or more doctor-diagnosed conditions were more likely to die from respiratory diseases than those who had no any health conditions. In addition, smoking was highly correlated with mortality caused by respiratory diseases. The odds of dying from respiratory cancer for current smokers were more than 20 times higher than the odds for those who never smoked. Significant associations were also found between the respiratory disease mortality and BMI, health limitation, alcohol consumption, and physical inactivity.

The results of the multinomial logistic regression model are summarized in Table 4. Older construction workers were almost twice as likely to die from respiratory cancer compared with their white-collar counterparts (OR = 1.65; 95% CI: 1.10–2.47), after adjusting for major demographics, health conditions, smoking, and other health behaviors. The odds of dying from NMRD were 73% higher for construction workers than white-collar workers in all industries (OR = 1.73; 95% CI: 1.16–2.58). The number of doctor-diagnosed conditions was a predictor for respiratory-cancer and NMRD -related mortality. The odds of current and former smokers dying from respiratory cancer were 21 times higher and seven times higher than the odds for never smokers, respectively.

## Discussion

Using a large nationally representative sample, this study found that construction workers were about twice as likely to die of respiratory cancer or NMRD compared to their white-collar counterparts, after adjusting for smoking and other major confounders. This study also showed that smoking elevated the risk of respiratory cancer and NMRD mortalities substantially among older workers.

Smoking is the number one risk factor for lung cancer and is linked to about 90% of lung cancer cases in the United States [35]. In 2010, nearly one in three construction workers were current smokers, compared to just 20% of workers in all industries [18]. The risk of chronic lung disease and lung cancer is magnified among construction workers due to the synergistic effects of smoking and other hazardous respiratory exposures, including welding dust, silica, and asbestos. This study provides clear confirmation of the risk of respiratory cancer and NMRD due to occupational exposures and smoking among construction workers.

This study also found that deaths from both respiratory cancer and NMRD were associated with a high number of diagnosed medical conditions and a low BMI reported one to two years prior to death. The majority of NMRD cases were “chronic lower respiratory diseases,” and were most likely COPD. Skeletal muscle dysfunction, nutritional abnormalities including weight loss, cardiovascular complications, metabolic complications, osteoporosis, and depression, are known associated ailments with COPD. These findings indicate that the association between respiratory cancer and multiple medical conditions or low BMI may be partially mediated by COPD, which is consistent with previous findings [36–38].

Strengths of this study include the use of a nationally representative sample, the detailed information on demographics, employment, smoking history, general health status, and chronic conditions, as well as the longitudinal nature of the HRS. In addition, cause of death among the study cohort was verified by the NDI data. Moreover, the adjusted odds ratios provide information on the independent impact of job exposure and smoking on respiratory-disease-related mortality among construction workers.

The primary limitation of the study is the sample size, which precluded analysis of respiratory cancer and NMRD within specific construction occupations. In addition, direct measures of respiratory hazards and occupational exposures were not available. The relationship between health indicators, such as BMI and respiratory cancer or NMRD, may reflect the severity of the underlying disease that led to death and may not necessarily represent a risk factor for the disease itself. Despite the limitations, this study confirms previous findings based on case studies and nonrandom samples, and adds to the growing evidence that respiratory cancer and NMRD are frequently caused by occupational exposures in the construction industry.

Taken together, the health and well-being of workers are greatly influenced by exposures to occupational hazards and risks associated with individual health behaviors [39]. The best way to protect workers from respiratory hazards is to have simultaneous prevention efforts against both occupational exposures and smoking. The National Institute for Occupational Safety and Health (NIOSH) has already integrated worksite health promotion with occupational safety and health interventions through the development of the Total Worker Health^™^ program, which can be used as a model for future prevention efforts.

## Figures and Tables

**Table 1:** Causes of mortality among workers 50 years and older, by longest occupation, 1992–2011.

Cause of Mortality (ICD-9 & ICD-10)	Construction			Other blue-collar			Service/Sales			White-collar		
	Number	Weighted number	Weighted %	Number	Weighted number	Weighted %	Number	Weighted number	Weighted %	Number	Weighted number	Weighted %
**Diseases of the circulatory system**	125	353,915	31.7%	596	1,651,713	34.2%	513	1,441,366	34.3%	658	2,090,555	34.3%
**Neoplasms/Malignant neoplasms**	111	361,272	32.3%	522	1,511,412	31.3%	367	1,137,405	27.1%	616	2,024,655	33.2%
Respiratory and intrathoracic organs	48	163,150	14.6%	188	517,864	10.7%	125	401,345	9.6%	178	507,057	8.3%
**Diseases of the respiratory system**	53	150,027	13.4%	189	557,674	11.5%	133	410,620	9.8%	172	540,328	8.9%
Chronic lower respiratory diseases	27	84,530	7.6%	101	304,349	6.3%	72	230,099	5.5%	100	328,032	5.4%
Lung diseases due to external agents	8	21,115	1.9%	13	28,492	0.6%	9	20,706	0.5%	6	21,002	0.3%
Influenza and pneumonia	6	16,458	1.5%	37	113,449	2.4%	29	84,132	2.0%	32	93,183	1.5%
All other and unspecified	12	27,924	2.5%	38	111,384	2.3%	23	75,684	1.8%	34	98,112	1.6%
**Endocrine/nutritional/metabolic diseases**	22	61,326	5.5%	71	183,914	3.8%	77	257,616	6.1%	62	173,000	2.8%
**Diseases of the nervous system**	16	31,828	2.8%	46	114,516	2.4%	40	118,210	2.8%	36	114,046	1.9%
**Diseases of the digestive system**	11	24,479	2.2%	63	182,874	3.8%	52	146,138	3.5%	101	275,519	4.5%
**Diseases of the genitourinary system**	10	39,989	3.6%	42	105,669	2.2%	57	190,977	4.5%	71	212,266	3.5%
**Other**	35	95,320	8.5%	185	525,873	10.9%	174	501,960	11.9%	221	671,887	11.0%
**Total**	383	1,118,156	100.0%	1,714	4,833,643	100.0%	1,413	4,204,293	100.0%	1,937	6,102,256	100.0%

Note: p-value < 0.001 reflects chi-square test for the association between causes of mortality and longest occupation

**Table 2:** Sample characteristics and respirator cancer- and NMRD-related mortalities by longest occupation and demographics, 1992–2011.

Characteristic	% of sample (n = 25,183)	Respiratory cancer-related mortality			NMRD-related mortality		
		Odds ratio	95% CI		Odds ratio	95% CI	
			Lower	Upper		Lower	Upper
**Longest occupation**							
Construction	6.3%	2.35	1.58	3.50	2.04	1.37	3.01
Other blue-collar	23.6%	2.04	1.59	2.62	2.03	1.59	2.59
Services/Sales	26.6%	1.31	1.08	1.61	1.26	0.94	1.70
White-collar	43.6%	1.00	1.00	1.00	1.00	1.00	1.00
**Longest job tenure**							
Mean (years)	18.9	18.3	17.2	19.4	21.8	20.4	23.2
≤5 years	10.3%	1.00	1.00	1.00	1.00	1.00	1.00
6–15 years	33.0%	0.95	0.67	1.35	0.88	0.68	1.15
>15 years	56.7%	0.91	0.68	1.21	1.06	0.79	1.41
**Age**							
Mean (years)	66.5	69.8	69.0	70.7	75.0	74.2	75.8
50–64	49.1%	1.00	1.00	1.00	1.00	1.00	1.00
65–74	28.4%	2.48	1.91	3.21	4.34	2.82	6.69
75+	22.6%	2.97	2.25	3.93	10.98	7.75	15.56
**Gender**							
Male	51.2%	1.47	1.22	1.77	1.52	1.27	1.82
Female	48.8%	1.00	1.00	1.00	1.00	1.00	1.00
**Race**							
White	82.8%	1.00	1.00	1.00	1.00	1.00	1.00
Black	11.2%	0.91	0.60	1.36	0.76	0.52	1.10
Other races	6.1%	0.63	0.31	1.29	0.43	0.26	0.72
**Hispanic origin**							
Hispanic	7.9%	0.45	0.25	0.81	0.41	0.24	0.72
Non-Hispanic	92.1%	1.00	1.00	1.00	1.00	1.00	1.00
**Education**							
High school or less	49.0%	3.87	2.60	5.76	5.21	3.54	7.66
Some college	25.0%	1.63	1.10	2.42	2.37	1.60	3.51
College and above	25.9%	1.00	1.00	1.00	1.00	1.00	1.00
**Marital status**							
Separated/Divorced	15.4%	1.35	0.97	1.88	1.67	1.27	2.21
Widowed	13.0%	2.16	1.64	2.85	3.72	3.01	4.61
Never married	5.7%	0.71	0.44	1.16	0.96	0.54	1.71
Married/Partnered	65.9%	1.00	1.00	1.00	1.00	1.00	1.00
**Geographic region**							
Northeast	17.7%	1.00	1.00	1.00	1.00	1.00	1.00
Midwest	24.5%	1.11	0.69	1.79	1.10	0.74	1.65
South	37.5%	1.27	0.84	1.92	0.86	0.63	1.20
West	20.1%	0.97	0.58	1.59	0.90	0.64	1.27

Note: Percentages are weighted. NMRD= non-malignant respiratory diseases

**Table 3:** Sample characteristics and respiratory cancer- and NRMD-related mortalities by health conditions and health behaviors, 1992–2011.

Characteristic	% of sample (n = 25,183)	Respiratory cancer-related mortality			NMRD-related mortality		
		Point estimate	95% CI		Point estimate	95% CI	
			Lower	Upper		Lower	Upper
**Physical health**							
Excellent/Very good	41.2%	1.00	1.00	1.00	1.00	1.00	1.00
Good	29.8%	2.04	1.42	2.94	2.37	1.66	3.40
Fair/Poor	29.0%	6.10	4.20	8.85	12.85	9.42	17.52
**Mental health (CES-D score)**							
Mean (score)	1.6	2.4	2.2	2.7	2.6	2.4	2.9
0	44.7%	1.00	1.00	1.00	1.00	1.00	1.00
1	21.4%	1.38	1.03	1.83	1.81	1.35	2.43
2	10.7%	2.43	1.71	3.44	2.68	1.87	3.85
3–8	23.1%	3.35	2.66	4.23	3.99	3.05	5.22
**Diagnosed health conditions**							
Mean (number)	2.0	2.7	2.5	2.8	3.2	3.1	3.4
0	17.1%	1.00	1.00	1.00	1.00	1.00	1.00
1–2	48.8%	2.21	1.45	3.39	5.91	3.10	11.27
3–8	34.0%	4.35	2.76	6.87	18.30	9.64	34.74
**Health limits work**							
Yes	33.2%	3.27	2.62	4.07	6.97	5.32	9.13
No	66.8%	1.00	1.00	1.00	1.00	1.00	1.00
**BMI**							
Underweight	1.0%	4.04	2.53	6.47	7.61	4.38	13.24
Normal weight	29.3%	1.00	1.00	1.00	1.00	1.00	1.00
Overweight	39.9%	0.55	0.44	0.68	0.47	0.37	0.60
Obese	29.7%	0.28	0.22	0.37	0.39	0.31	0.50
**Smoking status**							
Current smoker	17.3%	21.06	12.78	34.70	5.65	4.15	7.71
Former smoker	42.4%	9.61	6.37	14.48	4.61	3.62	5.89
Never smoker	40.3%	1.00	1.00	1.00	1.00	1.00	1.00
**Alcohol consumption**							
5+ drinks/day	3.4%	0.70	0.30	1.63	0.73	0.39	1.38
3–4 drinks/day	7.3%	0.89	0.63	1.26	0.31	0.18	0.52
1–2 drinks/day	29.4%	0.68	0.55	0.85	0.38	0.30	0.48
Non-drinker	59.9%	1.00	1.00	1.00	1.00	1.00	1.00
**Vigorous activity**							
Less than 3 times/week	73.3%	1.67	1.26	2.20	4.33	3.00	6.23
3+ times/week	26.7%	1.00	1.00	1.00	1.00	1.00	1.00

Note: Percentages are weighted. NMRD= non-malignant respiratory diseases

**Table 4:** Multinomial logistic regression of respiratory cancer- and NMRD-related deaths among older workers, 1992–2011.

Characteristic	Respiratory cancer-related deaths versus survivals			NMRD-related deaths versus survivals		
	Odds Ratio	95% CI		Odds Ratio	95% CI	
		Lower	Upper		Lower	Upper
**Longest occupation**						
Construction	1.65	1.10	2.47	1.73	1.16	2.58
Other blue-collar	1.57	1.23	2.00	1.68	1.30	2.18
Service/Sales	1.27	1.05	1.55	1.36	0.99	1.88
White-collar	1.00	1.00	1.00	1.00	1.00	1.00
**Longest job tenure**						
>15 years	0.89	0.65	1.23	0.80	0.56	1.15
6–15 years	1.00	0.70	1.44	0.87	0.63	1.20
≤5 years	1.00	1.00	1.00	1.00	1.00	1.00
**Age (years)**	1.05	1.03	1.06	1.09	1.08	1.11
**Gender**						
Male	1.36	1.08	1.71	1.77	1.37	2.30
Female	1.00	1.00	1.00	1.00	1.00	1.00
**Race**						
Black	0.90	0.60	1.34	0.78	0.55	1.11
Other races	0.87	0.41	1.87	0.76	0.47	1.24
White	1.00	1.00	1.00	1.00	1.00	1.00
**Hispanic origin**						
Hispanic	0.55	0.30	1.00	0.55	0.32	0.96
Non-Hispanic	1.00	1.00	1.00	1.00	1.00	1.00
**Marital status**						
Separated/Divorced	1.01	0.72	1.41	1.62	1.25	2.10
Widowed	1.41	1.06	1.88	1.71	1.36	2.15
Never married	0.73	0.44	1.20	1.16	0.62	2.19
Married/Partnered	1.00	1.00	1.00	1.00	1.00	1.00
**Sum of diagnosed conditions**						
0	1.00	1.00	1.00	1.00	1.00	1.00
1–2	1.99	1.30	3.04	4.47	2.33	8.58
3–8	3.51	2.19	5.62	10.31	5.37	19.81
**BMI**						
Underweight	3.23	2.02	5.17	7.8	4.51	13.46
Normal weight	1.00	1.00	1.00	1.00	1.00	1.00
Overweight	0.54	0.44	0.67	0.45	0.36	0.57
Obese	0.31	0.24	0.39	0.45	0.35	0.58
**Smoking status**						
Current smoker	20.51	12.38	33.97	6.90	4.71	10.10
Ever smoker	8.06	5.37	12.09	3.71	2.81	4.89
Never smoker	1.00	1.00	1.00	1.00	1.00	1.00
